# Zika Virus: Medical Countermeasure Development Challenges

**DOI:** 10.1371/journal.pntd.0004530

**Published:** 2016-03-02

**Authors:** Robert W. Malone, Jane Homan, Michael V. Callahan, Jill Glasspool-Malone, Lambodhar Damodaran, Adriano De Bernardi Schneider, Rebecca Zimler, James Talton, Ronald R. Cobb, Ivan Ruzic, Julie Smith-Gagen, Daniel Janies, James Wilson, Zika Response Working Group

**Affiliations:** 1 RW Malone MD LLC, Scottsville, Virginia, United States of America; 2 Class of 2016, Harvard Medical School Global Clinical Scholars Research Training Program, Boston, Massachusetts, United States of America; 3 ioGenetics, Madison, Wisconsin, United States of America; 4 Department of Medicine, Division of Infectious Diseases, Massachusetts General Hospital, Boston, Massachusetts, United States of America; 5 Department of Bioinformatics and Genomics, University of North Carolina at Charlotte, Charlotte, North Carolina, United States of America; 6 University of Florida, Department of Entomology and Nematology, Florida Medical Entomology Laboratory, Vero Beach, Florida, United States of America; 7 Nanotherapeutics, NANO-ADM Advanced Development and Manufacturing Center, Alachua, Florida, United States of America; 8 Analytical Outcomes, Washington Crossing, Pennsylvania, United States of America; 9 School of Community Health Sciences, University of Nevada, Reno, Nevada, United States of America; 10 Nevada Center for Infectious Disease Forecasting, University of Nevada, Reno, Nevada, United States of America; Colorado State University, UNITED STATES

## Abstract

**Introduction:**

Reports of high rates of primary microcephaly and Guillain–Barré syndrome associated with Zika virus infection in French Polynesia and Brazil have raised concerns that the virus circulating in these regions is a rapidly developing neuropathic, teratogenic, emerging infectious public health threat. There are no licensed medical countermeasures (vaccines, therapies or preventive drugs) available for Zika virus infection and disease. The Pan American Health Organization (PAHO) predicts that Zika virus will continue to spread and eventually reach all countries and territories in the Americas with endemic *Aedes* mosquitoes. This paper reviews the status of the Zika virus outbreak, including medical countermeasure options, with a focus on how the epidemiology, insect vectors, neuropathology, virology and immunology inform options and strategies available for medical countermeasure development and deployment.

**Methods:**

Multiple information sources were employed to support the review. These included publically available literature, patents, official communications, English and Lusophone lay press. Online surveys were distributed to physicians in the US, Mexico and Argentina and responses analyzed. Computational epitope analysis as well as infectious disease outbreak modeling and forecasting were implemented. Field observations in Brazil were compiled and interviews conducted with public health officials.

## Background and Introduction

Zika virus infection has spread rapidly in the tropical Americas since introduction to Brazil in 2014. Although a causal association is not yet confirmed, there is a growing consensus that Zika infection is linked to an upsurge in cases of Guillan Barré (GBS) syndrome and the birth of microcephalic infants following maternal infection [1, 2]. That association has become more likely with the publication of the report by Mlakar *et al* in which large numbers of viral particles were demonstrated in the central nervous tissue of an electively aborted microcephalic Zika-infected fetus [3].

The flavivirus Zika was first isolated from a *Rhesus* macaque obtained from the Zika forest of Uganda during 1947 [4, 5]. Zika virus is an enveloped, icosahedral positive strand RNA virus. The Zika virus reference genome (http://www.ncbi.nlm.nih.gov/nuccore/NC_012532.1) comprises a noncoding region and sequences coding for a 3419 amino acid polyprotein (http://www.ncbi.nlm.nih.gov/protein/226377834). Zika virus is related to yellow fever (YF), dengue, West Nile, and Japanese encephalitis viruses, and most closely to Spondweni virus [6, 7]. Studies in *Rhesus* macaque suggest that adaptive immune responses to Zika infection interfere with, but do not fully protect against, YF infection and disease [8, 9]. Serologic cross-reactivity, including non-neutralizing antibodies, is observed with other closely related flaviviruses and flavivirus vaccines.

Primates, including humans, are the best-documented Zika virus animal reservoir, with transmission to humans primarily by mosquito vectors (*Aedes spp*., including *Ae*. *aegypti* and *Ae*. *albopictus* [8, 10–13]. Soon after initial Zika virus discovery in Uganda, serologic evidence of human infection by Zika was observed in Egypt [14], India [15], Malaysia [15, 16], Thailand [16], Vietnam [16] and the Philippines [17]. Based on serology, but not verified by viral isolation, many other species may support Zika virus infection, including forest-dwelling birds [18], horses, goats, cattle, ducks and bats [19]. Recent reports indicate the potential for both human blood-borne and sexual transmission of Zika virus, including prolonged presence of virus in semen [20–23]. Zika virus is also present in the saliva of infected patients [24]. Perinatal transmission was documented in French Polynesia during the 2013–2014 outbreak where Zika virus sequences were identified in breast milk by polymerase chain reaction (PCR) [25], but reports from that outbreak did not indicate microcephaly as a complication. These observations underscore the need for more detailed studies to examine relationships between Zika virus pathogenesis, geography, and potential teratogenicity.

Historically, adult human infection with Zika virus has presented with mild, non-life threatening symptoms in 20% of infected patients, with 80% being clinically asymptomatic during initial infection. Typical acute symptoms persist from days to one week, and include fever (37.9°C or below), maculopapular rash (average duration 6 days), arthralgia (average duration 3.5d, range 1 to 14d) and/or conjunctivitis, myalgia, headache, retro-orbital pain and emesis. Based on blood bank screens in French Polynesia, it appears that viremia can begin up to 10 days before onset of symptoms, suggesting it may be longer than for some other arboviruses [20]. Recent reports of unusually high rates of GBS and primary microcephaly, which are temporally and spatially associated with the Zika virus outbreak in Brazil, have raised concerns that the virus variant circulating in these regions represents an altered public health threat, with neuropathic and teratogenic outcomes [26]. Death after Zika virus infection of an otherwise healthy patient with sickle cell disease has also been reported, indicating increased risk to otherwise medically compromised individuals [27]. The more severe Zika disease symptoms were not observed during the 2007 Yap Island, Micronesia, Zika outbreak, although approximately 5,000 people were infected [28]. Zika virus infection and disease is now a reportable illness in the United States, and as of February 2016, has spread to the countries and territories summarized in Table 1.

**Table 1:** Countries and territories with active Zika virus transmission.

American Samoa	Ecuador	Mexico
Barbados	El Salvador	Nicaragua
Bolivia	French Guiana	Panama
Brazil	Guadeloupe	Paraguay
Cape Verde	Guatemala	Saint Martin
Colombia	Guyana	Samoa
Commonwealth of Puerto Rico, US territory	Haiti	Suriname
Costa Rica	Honduras	Tonga
Curacao	Jamaica	U.S. Virgin Islands
Dominican Republic	Martinique	Venezuela

Source: [29]. See Fig 2 for additional details. As of February 17, 2016.

Clinical diagnosis of infection with Zika virus is complicated by similarities to other acute arboviral fevers, and Zika disease shares insect vectors and geographic range with dengue and chikungunya [30]. A case definition for Zika virus disease (“Zika”) has been developed by the World Health Organization [31]. A suspected case of Zika requires the presence of rash and/or fever with either arthralgia, arthritis, or non-purulent conjunctivitis. A probable case requires these symptoms in conjunction with the presence of anti-Zika IgM antibodies and an epidemiologic link within two weeks prior to symptom onset to a region with local autochthonous transmission. A confirmed case of Zika virus disease requires laboratory confirmation of recent Zika virus infection by either presence of Zika virus RNA or antigen in serum or other samples (e.g. saliva, tissues, urine, whole blood); or IgM antibody against Zika virus positive and PRNT90 for Zika virus with titre ≥20 and Zika virus PRNT90 titre ratio ≥ 4 compared to other flaviviruses; and exclusion of other flaviviruses.

GBS is a clinical syndrome of multiple autoimmune etiologies, which involve idiopathic peripheral neuropathy leading to acute flaccid paralysis [32]. Treatment consists of intravenous immunoglobulin and/or plasma exchange with supportive care for patients with respiratory compromise. The clinical course varies; 25% of patients require artificial ventilation (days to months), 20% of patients remain non-ambulatory at 6 months and 3–10% of patients die despite standard of care treatment. In medical care environments where ventilatory support is not readily available, GBS mortality is often much higher. Globally, annual GBS incidence is estimated at 1.1 to 1.8/100,000/year, of which approximately 70% appear associated with antecedent infectious disease. Such infections are typically gastrointestinal or respiratory, but include dengue infection [33–35]. A retrospective review of GBS cases (January 1995 through December 2002) at a São Paulo hospital documents an annual incidence of 0.6 cases/100,000/year, with a seasonal increase between September and March [36]. An abrupt surge in GBS, with significant mortality, is currently being observed in Brazil and other South American countries with Zika outbreaks. For example, during the 2015 rainy season, 50 of the 94 patients treated for GBS at the Hospital da Restauração in Recife, Brazil [37]. Retrospective seroneutralization analysis of GBS cases which were suspected of being associated with Zika during the 2013–2014 outbreak in French Polynesia has demonstrated that all 42 cases were positive for both dengue and Zika virus infection, yielding a ratio of 1 case of Zika-associated GBS for every 208 suspect cases of Zika virus infection [38]. However, the concomitant regional increase in dengue [39] and chikungunya [40] infections suggests that the increased GBS incidence may be attributable to these risk factors and/or to Zika infection.

Primary microcephaly (usually defined as head circumference ≤3 standard deviations below the mean at birth) is a rare multifactorial condition with incidence of from 1.3 to 150/100,000 live births (depending on consanguinity) [41]. Microcephaly is variously attributed to genetic factors, intrauterine infection (including rubella, toxoplasmosis, or cytomegalovirus), maternal malnutrition, and toxin exposure during gestation [42]. Symptoms include hearing loss, mental retardation, development delay, seizure disorders, and cerebral palsy. There is no specific treatment beyond supportive care. The reported annual incidence rate of microcephaly in all of Brazil was from 139 to 175 between 2010 and 2014 [43], or approximately 6/100,000 live births. The 3,530 cases of Zika-associated primary microcephaly reported in Brazil during 2015 yield a rate of 117/100,000 live births, indicating a twenty-fold increase in a single year. Retrospective review of French Polynesian birth data coinciding to the 2013–2014 Zika virus outbreak has confirmed that the incidence of central nervous system birth anomalies associated with that outbreak was well above average [44].

There are no specific licensed medical countermeasures (vaccines, therapeutics or preventive drugs) available for Zika virus infection and disease [45]. Diagnosis of Zika infection can be confirmed by PCR [46].

Clinical management of Zika is supportive and symptomatic, consisting of pain relief, fever reduction, and anti-histamines for the pruritic rash [26]. If a causal correlation between Zika virus infection and primary microcephaly and/or GBS is determined, rapid development of medical countermeasures to prevent and mitigate Zika-associated neurologic symptoms and birth defects.

We review and analyze information concerning the Zika virus outbreak in South America, Central America, and the Caribbean and the status of relevant medical countermeasures (MCM) available for treating or preventing Zika virus infection and disease. The analysis focuses on how the epidemiology, insect vectors, neuropathology, virology and immunology of this pathogen and outbreak inform options and strategies available for MCM deployment and future development.

## Methods

Multiple information sources were employed to support the review. These included publically available literature, including a review of peer reviewed journal papers and analysis of patent databases (Reuters, United States Patent and Trademark Office). Official bulletins and documents of the World Health Organization (WHO), European Center for Disease Prevention and Control (ECDC), United States Center for Disease Prevention and Control (CDC) were consulted, as well as statements on the websites of these agencies. The lay press of the English speaking and Lusophone world regarding the Zika virus outbreak was monitored. On-line surveys were distributed to physicians in the US, Mexico and Argentina and responses analyzed. Computational epitope analysis of Zika and comparative epitope analysis of Zika and related viruses and the human proteome was conducted [47]. Infectious disease outbreak modeling and forecasting was implemented [48]. Field observations in Brazil were compiled and interviews conducted with public health authorities.

## Discussion

### Summary of Findings

**Fig 1:**
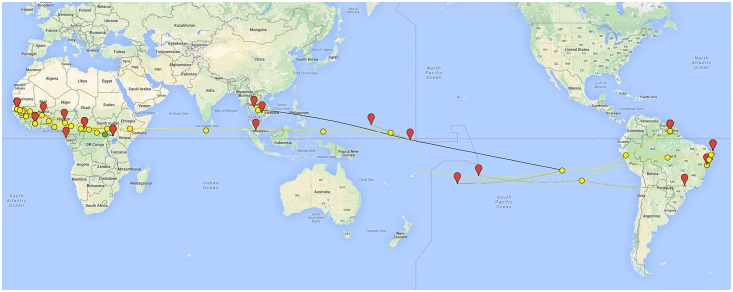
Phylogeographic analyses illustrating the lineage of the Zika virus currently circulating in Brazil. Phylogeographic analysis based on the envelope gene of Zika virus. This analysis illustrates the path of travel of Zika virus from Africa, Asia, and across the Pacific to South America. This analysis was created with Supramap [68]. Yellow circles and branches are associated with common ancestors. Red pins and black lines are associated with observed viral isolates. The root of the tree is indicated with a green circle. Data analyzed included all envelope variants of Zika virus available in the public domain as of January 18, 2016. Nucleotide sequence data were aligned using MAFFT v7.215 under default settings. A dataset for the envelope gene was created resulting in a matrix of 56 taxa and 753 aligned positions. A phylogenetic tree search was conducted for each dataset using RAxML v8.1.16 for 100 replicates under the GTRCAT model of nucleotide substitution. The outgroup was set to HQ234498. Supramap to project the phylogenetic tree into the earth [68].

Zika phylogenetic analysis indicates that the Zika virus lineage circulating in Brazil and Suriname shares common ancestry with viruses that have spread across the Pacific since 2007 (See Fig 1) [49]. While GBS was associated with the prior Polynesian outbreak [39, 50], the risk of pregnancy complications (teratogenicity) associated with Zika virus infections in the Americas may be substantially higher than previously reported [51, 52]. Primary clinical observations suggest that Zika-associated GBS in Brazil and South America follows typical symptoms, progression, and outcome risks associated with autoimmune GBS. While still unconfirmed, the increasing likelihood of a causal association between Zika infection, GBS and microcephaly demand that MCM development proceed with that expectation [44]. As the burden of the current Zika associated disease profile falls on neonates and their parents, the disability-adjusted life year (DALYS) cost impact will be very high.

Uncertainties about Zika virus transmission abound. The degree to which humans, non-human primates, or other animals can amplify and transmit the virus to insect vectors is poorly understood. The typical range and types of insect vectors observed in the past may not be predictive for the virus now circulating in the Americas. Infectivity of the circulating strain, viremia levels, duration, and risk of occult persistence are not yet understood.

The highest risk for introduction and establishment of autochthonous Zika transmission is likely to be associated with infected humans traveling by international ground, sea, and air transportation, and with the transport of mosquito larvae by trucks, ships and aircraft. Countering transportation-based introduction is the best immediate strategy available for delaying the spread. Options include more rigorous cargo fumigation at ports and border crossing points, use of larvicides and insecticides, and monitoring ground, sea, and air travel from infected areas. Seaports and the US/Mexico border are the most critical points for reducing the risk of large-scale vector borne viral distribution into the United States and Canada. Cases acquired abroad will continue to be identified in regions that have not reported autochthonous infection, and must be differentiated from local transmission. Rapid identification of infected persons who are subclinical and viremic is nearly impossible.

There is a critical need for development and deployment of Zika diagnostics to regional clinical reference laboratories (not just public health laboratories). Obstetricians throughout the Americas must advise their patients on very difficult decisions involving risk to ongoing or planned pregnancies. Neurologists are confronting unprecedented GBS outbreaks. These front-line physicians lack access to critical tests necessary to guide decisions, information concerning infection monitoring after possible exposure, understanding of the window of susceptibility to birth defects, and clear direction and resources for testing, diagnosing, and managing obstetric and neurology patients.

Delaying spread of the virus into new regions may buy some time to develop MCM, but will not help those who live in infected areas. During gestation, women of means may choose to leave infected countries for safe zones [53, 54]. The governments of several affected countries are recommending that pregnancies be deferred for up to two years for those who remain there [55]. Altered birth cohort progression throughout the region, coupled with disabled care, may have long-term disruptive political, systemic and economic impacts in these countries.

In affected areas, regional surges in GBS may stress medical response capacity. MCM preparation for GBS surges should include sufficient intensive care unit capacity, ventilators, plasma exchange equipment [56], trained support personnel, and intravenous immunoglobulin (IVIG) [57, 58]. Regional IVIG supplies in affected areas may be at risk due to a combination of high demand and reduced availability secondary to blood donation restrictions designed to limit virus transmission *via* blood products. Procedures for minimizing risk of salivary transmission must be developed [24]. Guidance concerning blood bank risk management has recently been established, and must be promptly implemented [20, 59, 60]

### The Evolving Epidemiology of Zika Virus Spread into the Americas

In contrast to the relatively slow spread of Ebola virus through West Africa, the Zika outbreak in the Americas appears to be moving very rapidly. While the potential association of Zika virus with teratology and neuropathology place a particular urgency on the development of MCM, strategies for developing and deploying MCM must account for the differences and similarities between the observed epidemiology and that of prior outbreaks. For example, developing, testing and deploying a new vaccine may be feasible for endemic pathogens or slowly moving epidemics, but may not be practical for a rapidly moving infectious disease outbreak. Until the pathogenesis of the disease, nature of vectors and mechanisms of spread are understood, caution must be exercised in making assumptions in the design of MCM.

Flaviviruses can appear significantly more pathogenic when introduced into new niches and populations, but as a new virus becomes established, herd immunity effects often attenuate apparent virulence. West Nile virus in birds shifted from a relatively benign profile in the traditional endemic African host range to very high mortality upon introduction in North America in 1999. This change was associated with specific mutations that increased viral reproductive fitness in avian hosts and the North American environment [61]. The rapid spread of chikungunya, an unrelated alphavirus, into India was the result of adaptation to a different mosquito vector resulting from a single nucleotide change [62]. The patterns of rapid evolutionary radiation of these arboviruses into new niches, and their associated pathophysiology, may help inform hypothesis development concerning patterns of infection and disease in the Zika virus outbreak in the Americas.

Many questions about Zika virus epidemiology and transmission remain, but among the most pressing questions are whether the change in disease phenotype correlates to changes in viral genotype, and if current clinical disease is influenced by viral entry into a new population with indigenous confounding or effect modification. Historically restricted to Africa and Asia, outbreaks of autochthonous Zika virus infection were reported in Micronesia beginning in 2007 [7, 28]. As predicted by Hayes [63], widespread autochthonous outbreaks of Zika virus were then reported in French Polynesia in October 2013 [64], New Caledonia in January 2014 [64], Cook Islands in February 2014 [64], and Easter Island in February 2014 [65]. Zika then began to infect patients in South America in 2014 [66]. The first molecularly confirmed case of Zika virus infection in Brazil was identified in March 2015 [67].

To summarize these events and to help guide assessment of genetic and immunologic differences between historic and current Zika virus populations, we performed preliminary phylogeographic analyses of available molecular sequence data and metadata (place and time of isolation) from the viruses to connect these incidents *via* shared ancestry of the sequences. Based on data released as of January 18, 2016 we have focused on two genes to reconstruct the spread and evolution of Zika from Africa to Southeast Asia to the South Pacific and to South America; E (Envelope) and NS5 (RNA-dependent-RNA-polymerase). Results from applying this method for tracking and summarizing sequence accessions, together with associated temporal and geographic metadata, suggest a pattern of stepwise accumulation of sequence changes. The Zika virus circulating in the Americas appears to have acquired mutations while hopping along distant points across the Pacific, and then emerged as a burst of infection by a cohort of closely related viruses upon arrival in Brazil.

The initial introduction of Zika virus into continental South America may have occurred in Brazil during 2014 or very early 2015. Our results suggest entry to Brazil from the Cook Islands (as suggested by analysis of the E gene) or Easter Island (as suggested by analysis of the NS5 gene). Some speculation concerning viral introduction into Brazil near Rio de Janeiro has assumed that the virus was imported by infected humans, and has centered on two sporting events which included participants from Polynesia (the 2014 FIFA World Cup and the Va’a World Sprint Canoe World Championships) [69]. These sporting events occurred during June, July and August of 2014. Other Brazilian researchers question this hypothesis, noting that data suggests an original epicenter in the Brazilian northeast [70] (states of Rio Grande do Norte, Bahia, and Pernambuco). Our preliminary phylogeographic analysis is consistent with both of these hypotheses. Additional annotated sequence data may enable more precise assessment of the likely entry point and time.

After introduction, Zika virus rapidly spread throughout much of Brazil. In January 2016, there were cases in 14 states in Brazil [71] and in neighboring countries including Colombia and Venezuela [44, 72] Zika cases have also been recently reported in Cape Verde, but molecular data necessary to assess whether they are linked to South America or Africa is not yet available [73]. Similarly, there were no molecular data in the public domain for Zika cases in Central America, the Caribbean, and Mexico as of January 2016.

As summarized in Table 2, the Brazilian Ministry of Health has estimated that between 440,000 and 1,300,000 cases of Zika virus infection may have occurred in Brazil during 2015 [71]. These numbers, which have served as the primary estimate of Zika incidence in Brazil for ECDC and other public health analyses, must be recognized as a best estimate rather than actual incidence data. Therefore, all epidemiologic analyses of rates and relative risks are based on this best estimate of the range of overall incidence in the affected states of Brazil, and on the reported and verified cases of Zika associated primary microcephaly in Brazil at large. The underlying estimates of incidence are likely to change as additional data become available, and epidemiologic summary statistics will change as these estimates are refined.

**Table 2:** Projection of Zika virus infections in states with laboratory confirmation of Zika virus circulation during 2015 (18 of 27 Brazilian states or federated units).

Brazil	Estimated Zika Virus Infections		Brazil	Estimated Zika Virus Infections	
Federated unit	Lower limit	Upper Limit	Federated unit	Lower limit	Upper Limit
Alagoas	4,023	29,066	Paraná	42,008	97,118
Amazonas	3,119	34,264	Pernambuco	34,579	81,303
Bahia	19,216	132,274	Piauí	3,237	27,875
Ceará	38,485	77,469	Rio de Janeiro	15,918	143,985
Espírito Santo	6,481	34,190	Rio Grande do Norte	4,761	29,947
Maranhão	1,481	60,067	Rondônia	2,911	15,383
Mato Grosso	8,202	28,410	Roraima	1,450	4,399
Pará	6,357	71,400	São Paulo	236,494	386,249
Paraíba	6,013	34,558	Tocantins	8,767	13,182
Brazil				443,502	1,301,140

The parameters utilized for this estimate were developed by employing dengue case frequencies for the inferior limit and the proportions of cases that occurred in French Polynesia for the upper limit based on the population in each state. These speculative values are an estimate of the dispersion potential of this virus, which has over 80% asymptomatic or oligosymptomatic cases (translated from Portuguese). See reference: [74].

Although human transmission may be a source of initial introduction into Brazil in 2014, the apparent incidence of new infection in the region implies a high reproduction number (R_0_). Other means of introduction must also be considered, including birds or insects *via* cargo shipping. Evidence supporting avian infection by Zika virus has been reported [18], but the prevalence in birds and potential of transmission from avian species to humans *via* insect intermediates has not been studied. West Nile virus was rapidly spread throughout North America by birds. Transoceanic movements of arboviruses in insects has been reported [75]. However, the relative absence of Zika along the western coast of South America argues against wind or avian-borne introduction across the Andes into northeastern Brazil from Polynesia or Easter Island. The greatest potential for new introduction and establishment of local autochthonous transmission appears to be a combination of viremic human importation by ground, sea and air, and/or cargo-associated transport of infected mosquitos and larvae by trucks, ships and airplanes. Therefore, countering human and freight-based introduction appears to be the best countermeasure strategy available for delaying the spread of the Zika virus into new regions of the Americas. Data demonstrating that human viremia precedes clinical symptoms suggests that screening by symptoms at points of entry may be problematic [20].

In the case of the West African Ebola outbreak of 2014 to present, rapid communication, adoption of effective outbreak tracing and control measures, and cultural changes reduced transmission to the point that vaccine trial efficacy endpoints could not be met. In the preceding Zika outbreak on Yap island in Micronesia, the overall attack rate observed for confirmed and probable Zika virus disease among patients presenting to health care facilities was 14.6 per 1000 Yap residents (range of 3.6 to 21.5 per 1000 population). During what appears to have been a four month, self-limited outbreak, it is estimated that 73% of Yap residents 3 years of age or older were infected with a Zika virus strain hypothesized to have been brought to the island by an imported non-human primate [28]. This suggests that, with the current Zika outbreak, the virus may spread so efficiently that by the time a vaccine becomes available to test in human clinical trials, identifying large naïve at-risk populations may be an obstacle to demonstrating efficacy.

**Fig 2:**
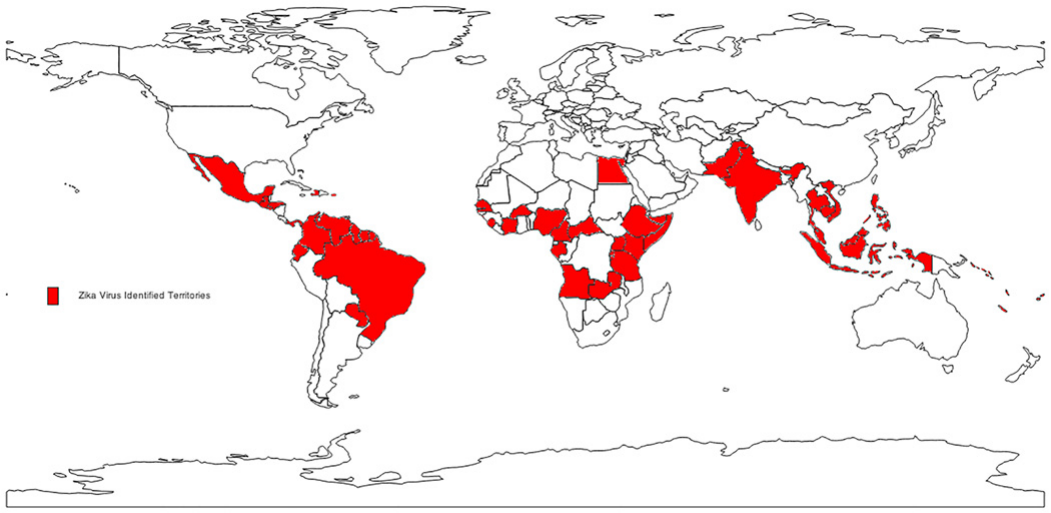
Zika virus, past and current distribution. Source: Centers for Disease Control and Prevention [83].

Zika virus evolution and spread is constrained by both human and insect hosts, and this creates an opportunity to develop countermeasure strategies focusing on either or both. The interaction between pathogen and host biology will impact the incidence, prevalence and eventual distribution of the virus. As Zika adapts to new niches in the Americas, the roles played by humans and non-human primates, other animals and arthropods as primary and intermediate hosts must be understood. Factors which will influence the rate of spread include availability of vector species, temperature and humidity available to support transmissibility, and high mosquito to human contact rates. Similar to dengue and chikungunya, *Aedes* sp. (*Ae*. *aegypti* and *Ae*. *albopictus*) appear to be the leading candidate Zika vectors in the outbreak. Potential involvement of other insect vectors including *Culex* sp. mosquitoes are currently being examined [76, 77]. In the outbreak on Yap island, 12 mosquito species belonging to four genera were identified as potential vectors, and *Ae*. *hensilli* Farner was the predominant vector species [28]. The distribution of *Ae*. *aegypti* and *Ae*. *albopictus* mosquito populations reaches around the globe, with remarkable parallels to the global distribution of Zika virus (Fig 2). *Ae*. *aegypti* populations are predominately located in the subtropics and tropics. In contrast, *Ae*. *albopictus* is able to survive cooler temperatures and has high ecological plasticity. *Ae*. *albopictus*, is distributed through the northern United States, southern Brazil, northern China, and southern Europe, as well as Africa, Central America, and Australia [78, 79], and is rapidly colonizing new regions. This territory expansion is aided by temperature changes, globalization and urbanization [78, 79]; all factors which are also associated with increased risk of autochthonous Zika virus transmission. Improved understanding of the vectors involved may help explain the outbreak, and must guide the public health response [78, 80]. For example, *Ae*. *aegypti* and *Ae*. *albopictus* are both widely distributed in the United States [78]. Due to greater cold tolerance, *Ae*. *Albopictus* could spread the virus further into the North East and Midwestern US, and perhaps Canada (see Fig 2). In Africa, the virus has been isolated from a wide range of *Aedes* species [13]. Therefore, it will be important to understand which species can carry Zika in Latin and the Caribbean, and whether other *Aedes* species, or other vector species, present any risk in North America. Ultimately, the distribution of the virus will be determined by the distribution of competent insect vectors and the strategies developed to interfere with the virus-vector cycle.

Predictions of an unusually severe *El Niño* weather pattern favoring mosquito reproduction, coupled with the pending 2016 Rio de Janeiro Summer Olympic games [81] and well established cargo and cruise shipping routes between South America, the Caribbean, Gulf of Mexico and Eastern seaboard ports in North America suggest the potential for further spread of Zika virus during 2016 to many regions of the Americas which support *Ae*. *aegypti* and *Ae*. *albopictus* mosquito populations, including significant portions of the continental United States. PAHO predicts that all countries in the Americas where *Aedes* mosquitos are found will eventually become infected with Zika virus [82].

### Zika Neuropathology and Teratology

In other recent outbreaks, Zika disease has been subclinical or mild [28]. What makes this outbreak a high priority global public health concern is the association with incidence of birth defects involving the central nervous system and the apparent increased incidence of GBS. The immediate need is for MCM to treat Zika-associated GBS and other neuropathy [84] in the adult, and to prevent the teratogenic outcomes which may be collectively referred to as Zika fetal syndrome (primary microcephaly [3], retinopathy [85, 86], and other neurologic birth defects). To optimize MCM development, the link between infectious cause and clinical effect must be clearly established. However, as evidence has accumulated, skepticism about a causal link between Zika spread and primary microcephaly incidence has given way to growing acceptance that Zika virus infection during the first and second trimester may be a major contributing factor to the surge in microcephaly. The possible increase in GBS incidence, associated morbidity and mortality, and potential association of these disease symptoms with Zika is not as solid. Interpretation of any change in overall GBS incidence in the region attributable to Zika virus is complicated by local fluctuations in the incidence of dengue and chikungunya [87].

When applying Bradford Hill’s criteria for establishing epidemiologic causation to the current Zika virus outbreak [88], the most obvious paradox is why a possible correlation between Zika infection, microcephaly and GBS was not detected in outbreaks prior to the 2013–2014 French Polynesian experience [89, 90]. This appears to violate the requirement for consistency, but may indicate the variable presence of another risk factor in addition to Zika virus. The apparent lack of consistency may reflect an interaction between host and/or viral genetics and the environment, or the presence of one or more additional risk factor variables [88]. Since the more severe outcomes observed (GBS and Zika fetal syndrome) may have an autoimmune component, it may not be necessary for each risk factor to be concurrent. The specific pathogen(s), potential confounders or effect modifiers, and the mechanistic basis of the GBS and central nervous system teratogenicity observed in this outbreak must be better understood.

Public health awareness of a possible link between the Zika virus outbreak and microcephaly gradually developed during the second half of 2015. Reports of an unusual increase in the number of children born with microcephaly in 2015 in the Brazilian state of Pernambuco, followed by analysis of data from the Brazilian live birth information system (SINASC), documented a significant increase in the number of microcephaly cases compared with previous years. Temporal and spatial concordance of the distribution of primary microcephaly with that of Zika virus infection raised public health concerns of a possible causal relationship [91]. These findings led to a November 11, 2015 declaration of a public health emergency by the Brazilian Ministry of Health [92]. Assuming initial viral entry into Brazil sometime during June-August of 2014, this timeline is consistent with a causal relationship.

The link between high rates of microcephaly and Zika infection was initially greeted with skepticism. Although a possible link between microcephaly and Zika virus infection was first reported in French Polynesia (the apparent source of the virus which seeded Brazil), it has not been reported over the many prior years that Zika has existed in its traditional endemic range [63, 93, 94]. While this may be the result of a case (under) reporting phenomenon, it is more plausible that girls in endemic areas are infected and become immune well before childbearing age. Therefore, one hypothesis is that the outbreak of microcephaly in Brazil is the consequence of recent introduction to a fully susceptible population, including pregnant women. There are examples of arbovirus-caused teratology in domestic animals at the leading edge of vector borne incursion [95]. Understanding the age of infection in endemic areas and whether childhood exposure provides protection could help clarify the paradox of low microcephaly rate in endemic regions, and would guide immunization strategy when a vaccine becomes available. An alternative hypothesis is that viral evolutionary changes have given rise to a new spectrum of Zika disease.

Koch’s postulates concerning infectious disease causality include demonstrating the presence of the pathogen in affected patients. Molecular biologic evidence demonstrating Zika genomes in tissue and amniotic fluid of Brazilian children born with microcephaly “support the conclusion of the rapid risk assessment of 24 November that a causal association between microcephaly in newborns and Zika virus infection during pregnancy is plausible” [26]. More recently, a small Brazilian case-series describing intrauterine transmission of Zika in humans has been published [96, 97]. In this study, sequencing of viral nucleic acids obtained *via* amniocentesis confirmed presence of Asian-type Zika virus. Ultrasound analysis revealed findings similar to those observed with cytomegalovirus infection (but more severe), and also similar to those previously reported with intrauterine infection by West Nile virus. On the basis of observed ultrasound findings, the authors of the alert speculate that “as with other intrauterine infections, it is possible that the reported cases of microcephaly represent only the more severely affected children and that newborns with less severe disease, affecting not only the brain but also other organs, have not yet been diagnosed.” This has since proven true with the reports of ocular lesions in affected infants [85]. A recent report of a case imported into Europe from Borneo provided electron microscopy evidence of Zika–like virions in a fetus from a terminated pregnancy [3]. To our knowledge, however, there have not yet been replicating virus isolates obtained from affected fetuses or placental tissues, although full length viral genome has been recovered [97].

Concurrent with growing evidence from Brazil of a correlation between Zika infection microcephaly, on November 24, 2015 French Polynesian public health authorities published a report documenting an increase of at least 17 cases of primary microcephaly relative to background incidence during 2014–2015. Based on the timing of the Zika outbreak in French Polynesia, this report hypothesizes peak sensitivity to teratogenic effects during the first or second trimester [91]. These findings, supported by evidence indicating an increased incidence of GBS syndrome in patients infected with Zika virus, were sufficient to lead PAHO to issue a public health alert on December 1, 2015 concerning potential associations between neurological syndromes, congenital malformations, and Zika virus infection [98].

**Fig 3:**
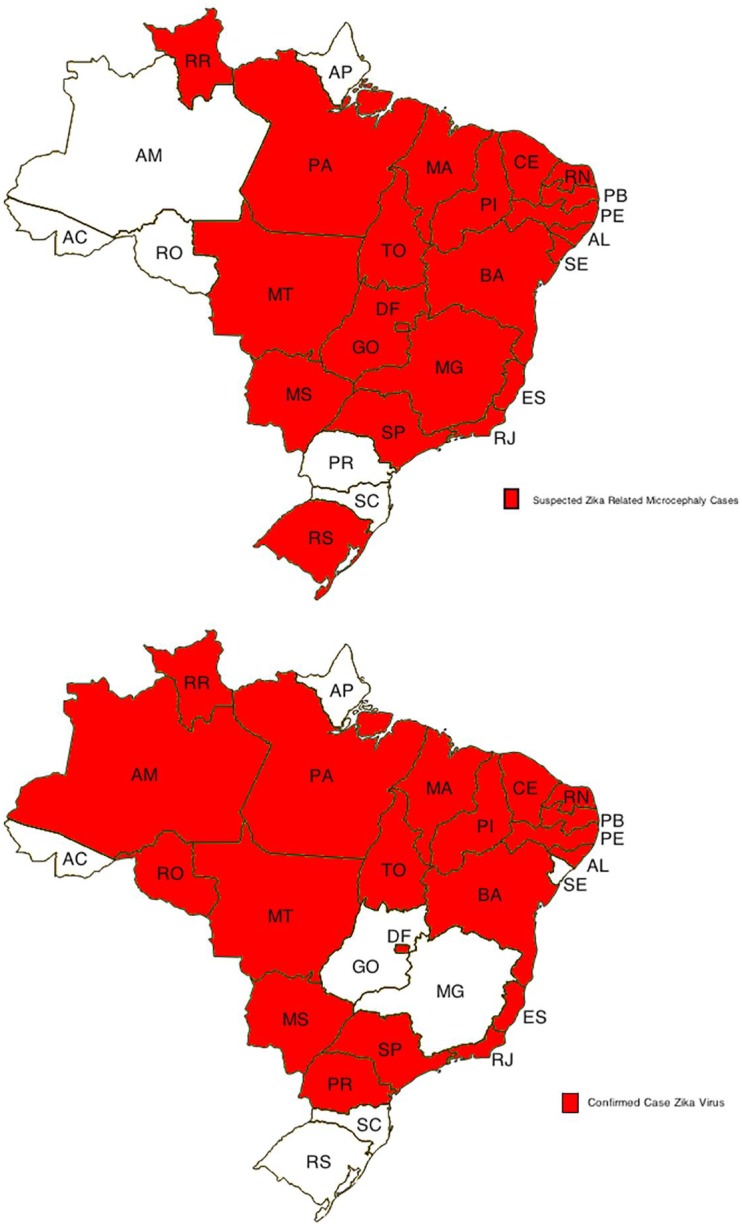
States in Brazil investigating microcephaly cases for association with Zika virus infection (above), and with confirmed circulation of Zika virus (below). After [99]. Information sources include Brazilian Health Ministry (Ministério da Saúde);WHO (World Health Organization); PAHO (Pan American Health Organization).

Strength of epidemiologic association and evidence indicating a biological gradient correlating exposure and disease are also key criteria for establishing causation [88]. Fig 3 illustrates the distribution of Brazilian states currently investigating an association between primary microcephaly cases and Zika infection, and those currently reporting circulation of Zika virus, with additional detail being provided in Table 3.

**Table 3:** Summary of Brazilian States (Federated units), current Zika circulation patterns, and increased incidence of primary microcephaly.

Brazilian State		Zika Circulation	Primary microcephaly	Brazilian State		Zika Circulation	Primary microcephaly
Acre	AC			Pará	PA	+	+
Alagoas	AL	+	+	Paraíba	PB		
Amapá	AP			Paraná	PR	+	
Amazonas	AM	+		Pernambuco	PE	+	+
Bahia	BA	+	+	Piauí	PI	+	+
Ceará	CE	+	+	Rio de Janeiro	RJ	+	
Distrito Federal	DF	+	+	Rio Grande do Norte	RN	+	+
Espírito Santo	ES	+	+	Rio Grande do Sul	RS		+
Goiás	GO		+	Rondônia	RO	+	
Maranhão	MA	+	+	Roraima	RR	+	+
Mato Grosso	MT	+	+	Santa Catarina	SC		
Mato Grosso do Sul	MS	+	+	São Paulo	SP	+	
Minas Gerais	MG		+	Sergipe	SE		+
				Tocantins	TO	+	+

After [99]. Information sources include Brazilian Health Ministry (Ministério da Saúde); WHO (World Health Organization); PAHO (Pan American Health Organization).

The state of Pernambuco (located in northeastern Brazil) was the first to identify an increase of microcephaly, and has reported 1,236 cases up to January 09 (35% of total), followed by Paraíba (569), Bahia (450), Ceará (192), Rio Grande do Norte (181), Sergipe (155), Alagoas (149), Mato Grosso (129) and Rio de Janeiro (122) [100]. In the northwestern region there were 46 additional microcephalic neonatal deaths being investigated for Zika virus involvement as of January 15, 2016. Considering the average annual birth rate in Brazil of 1.5% [101], this would indicate 6,600 to 19,500 pregnancies at risk of primary microcephaly from Zika virus infection. On average, in Brazilian states reporting Zika infection during 2015, the attack rate for 2015 is estimated to have been between 0.30% and 0.88%. These numbers yield an annual cumulative incidence rate estimate for Brazilian mothers infected with Zika during pregnancy delivering infants with primary microcephaly ranging from 18% to 53%. Based on these best estimates of overall Zika incidence, Brazilian mothers infected with Zika during pregnancy are between 3,700 to 11,000 times more likely to deliver infants with primary microcephaly compared to uninfected mothers. Table 4 provides a comparison of predicted versus reported cases of microcephaly (derived from the data summarized in Table 2). This summary suggests that the verified cases of microcephaly in Brazil may under-represent the actual incidence between October 22, 2015 and January 09, 2016. These data appear to indicate epidemiologic association of Zika virus and microcephaly, as well as a correlation between gradient of Zika exposure and microcephaly. However, they do not provide a mechanism for the pathogenesis.

**Table 4:** Comparison of predicted to reported cumulative case incidence distribution of primary microcephaly by federated unit (state), Brazil, 2015.

Brazil	Reported cases	Predicted Cases		Brazil	Reported cases	Predicted Cases	
Federated unit with Zika		Lower Limit	Upper Limit	Federated unit with Zika		Lower Limit	Upper Limit
Alagoas	149	32	78	Paraná	No data	334	262
Amazonas	No data	25	93	Pernambuco	1,236	275	220
Bahia	450	153	357	Piauí	No data	26	75
Ceará	192	306	209	Rio de Janeiro	122	127	386
Espírito Santo	No data	52	92	Rio Grande do Norte	181	38	81
Maranhão	No data	12	162	Rondônia	No data	23	42
Mato Grosso	129	65	77	Roraima	No data	12	12
Pará	No data	51	193	São Paulo	No data	1,880	1,043
Paraíba	569	48	93	Tocantins	No data	70	36
Brazil (18 of 27 states reporting)						3,526	3,515

Table based on estimates provided by Brazilian Ministry of Health as summarized in Table 2. Numbers of predicted cases are derived by calculating predicted at-risk pregnancies (the product of average crude birth rate in Brazil between 2011–2013 of 15 births/1000 people and estimated Zika infected population in each state summarized in Table 2) and multiplying by the corresponding calculated average incidence rate estimate lower and upper limits for the country at large during 2015.

### Zika Virology and Immunology

In the typical initial infection event, Zika virus is transmitted to a bitten human host after skin injection of a mixture of insect saliva, virus, and blood components from the most recent feeding during female mosquito blood meals. Probability of viral particle transmission is related to the volume of fluid held in the proboscis from a prior blood meal, viral replication levels and volume of insect salivary glands, and the viral infectious titer of the preceding host [102]. In the case of many arboviruses, mosquito salivary gland products enhance viral infectivity and replication. Zika infection of the recipient host requires viral envelope protein binding and particle uptake into susceptible cells, is mediated by specific receptors which include DC-SIGN, AXL, Tyro3, and TIM-1, and triggers transcriptional activation of Toll-like receptor 3 (TLR3), RIG-I, MDA5, interferon stimulated genes including OAS2, ISG15, and MX1, and beta interferon [103]. Primarily infected cells include skin fibroblasts, epidermal keratinocytes, and skin dendritic cells. Immature dendritic cells appear to be an important initial Zika target. Reasoning by analogy to dengue infection, it is likely that primary Zika infection triggers apoptosis of infected cells, thereby evading aspects of innate immune responses and increasing initial release of infectious viral particles [102]. Both dengue and Zika viruses subsequently exploit autophagy to enhance replication [104], and pharmacologic manipulation of Zika-infected cells with 3-Methyladenine (3-MA), an inhibitor of autophagosome formation, strongly reduces viral copy numbers in infected fibroblasts [103]. Based on prior murine studies involving Zika virus inoculation in mouse brain [105], autophagy of Zika virus has been postulated as playing a key role in the pathogenesis of Zika-associated primary microcephaly [106].

The infection and host response cascade triggered by initial infection with Zika virus has yet to be characterized. Dengue infection in humans may provide a model until further information becomes available. In the case of dengue, the infection then spreads to both lymphatic and non-lymphatic tissues; fever, arthralgia and myalgia ensue. Viral titers peak with fever onset, are stable for one to two days, and then decline as adaptive immune responses begin to control the infection (T and B cells), with IgM and IgG levels increasing rapidly as viremia drops. The CD8+ T cell responses to dengue infection are primarily directed to nonstructural protein epitopes including NS3 and NS5. Human infection by dengue provides one of the most classic examples of antibody dependent enhancement of disease by pre-existing non-neutralizing antibody, resulting in dengue hemorrhagic fever [107, 108]. The potential role of antibody dependent enhancement (ADE) of Zika infection and disease has not been examined.

The duration of viremia, infectivity, and persistence of Zika virus, is not known for either post-partum or intrauterine infection. Nor is the route of fetal infection, or the degree of neurotropism. Related flaviviruses may cause persistent infection despite the presence of serum antibodies [109]. West Nile virus can be neurotropic in many species including humans [110, 111]. Dengue is associated with encephalitis, encephalopathy, and multiple less frequent neurological symptoms [33, 35]. Transplacental transmission of West Nile virus has been reported [112]. Dengue infection in pregnancy leads to transplacental transfer of anti-dengue antibodies [113–115]. However, despite the extensive distribution of dengue, there is only one published case study showing transplacental fetal infection[116]. Zika virus has been demonstrated in amniotic fluid [97, 117], as well as in an aborted fetus [3]. Researchers from the Carlos Chagas Institute of Paraná Fiocruz have reported that Zika virus can cross the placenta during pregnancy, based on demonstration of viral proteins in placental cells. The working hypothesis offered for the Zika viral transplacental transport mechanism is that the virus may be using the migratory capacity of these cells to reach fetal vessels [118]. An alternative explanation for Zika virus infection of amniotic fluid and, possibly, fetal central nervous tissue may be viral uptake and transport *via* FcRn receptors on the placenta. Epitopes with dengue or YF could result in preexisting antibodies to these viruses binding Zika and enhancing initial virus replication or placental cell infection, or transplacental viral transfer.

Rapid immunoinformatic analysis of the envelope protein of Zika, from Brazilian Zika SPH2015 (KU321639), indicates predicted B and T cell epitopes in peptides that are consistent to those reported for dengue, YFYF and Japanese encephalitis. The envelope Domain II B cell epitope, to which much dengue non-neutralizing cross reaction is attributed [119], is also conserved also in Zika, consistent with prior field observations of cross reactivity with dengue and YF. Domain III of the Zika envelope protein, likely the main specific neutralizing domain, is distinct from recent Brazilian dengue isolates. When compared with recent Brazilian dengue 1–4 isolates (GQ330473, HQ184924, JF808120, JN848496, JQ513335, KP858105, KP858119, HQ184925, JN848499, KP858111) and a recent Peruvian YF isolate (GQ379163), 76% of possible major histocompatibility complex class (MHC) I and MHC II binding peptides and potential B cell linear epitopes are unique to Zika. Related to this, the patterns of similarity of T and B cell motifs with the human proteome differs in Zika relative to dengue, indicating a potentially different pattern of epitope mimics. When envelopes of 38 strains of Zika from around the world are compared [13, 120], the Cook Island and Brazilian isolates stand apart from two clusters of African isolates, based on analysis of B cell linear epitopes and predicted MHC II binding.

Opportunities and strategies for Zika medical management and countermeasure development will benefit from answers to key questions concerning the virology and immunology of Zika infection in the human host. A better understanding of natural immune responses and viral infection may clarify the potential role of Zika in eliciting GBS or microcephaly. Targeted identification and design of antivirals, neutralizing antibody preparations and immunotherapeutics still require understanding of the underlying biology. Critical priorities for early characterization include duration and levels of viremia and transmissibility, whether circulating non-neutralizing antibody complexes contribute to either primary infection or fetal pathology, and the potential for interaction with pre-existing immunity elicited by other flaviviruses or flavivirus vaccines.

### Medical Countermeasure Development Strategies

Over the short term, development and testing of antiviral drugs, neutralizing antibody preparations, and medicines designed to interfere with Fc receptor interactions [121] are among many MCM strategies which must be evaluated for those at greatest risk—pregnant women in their first and second trimesters [122, 123]. Product candidates with antiviral potency can be rapidly selected and evaluated using *in vitro* tests and animal challenge models. Once identified, testing of medical products may be expedited by focusing on high-risk populations (pregnant women and those wishing to become pregnant); risk/benefit ratios in these populations may be more compelling, and clinical safety and efficacy testing may be more efficient when subpopulations with higher risk for clearly defined disease outcomes, rather than general populations, are selected for clinical study enrollment. Pregnant women are typically the last “special population” to be clinically tested when developing a MCM, but this outbreak represents a special case where the fetus is apparently at highest risk.

Development of a general use prophylactic vaccine for Zika virus-induced disease will require considerable time and careful evaluation of safety, effectiveness, and risk/benefit ratio for the population at large. This is particularly true for a vaccine designed to protect against a virus apparently associated with both neurologic teratogenic effects and neurologic autoimmune disease (GBS), and which belongs to a genus notorious for antibody-mediated enhancement of infection [107, 124, 125]. For example, during 2002 it was announced that a vaccine for the closely related West Nile Virus was in preparation with licensure anticipated within three years [126]. While an equine vaccine for West Nile Virus has been licensed, there are currently no vaccines licensed for preventing West Nile Virus disease in humans. With any prophylactic vaccine intended for human use, the requirement for careful evaluation of safety (including potential for eliciting autoimmune disease) and efficacy necessitate large and sustained clinical development efforts [127–130]. In Brazil, Institute Butantan has announced an expedited Zika vaccine development effort projected for completion in three to five years after an initial year of non-human primate testing, which may involve collaboration with the NIH [131]. Experience suggests that this is an optimistic timeline for development and licensure of a flavivirus vaccine, which may require up to twenty years of clinical development and testing [132].

In the Yap island outbreak of 2007, 73% of the residents of Yap were infected by Zika within four months [28]. By the time marketing authorization is granted for a general use prophylactic vaccine, Zika may have become endemic in susceptible regions of the Americas, with a large fraction of the population having become infected during childhood or adolescence. Hopefully such infection will provide subsequent protection from both adult GBS and transplacental infection, as appears may be the case in other endemic regions. However, this scenario offers little solace for the patients, parents (and would-be parents), primary caregivers, obstetricians, neurologists and public health officials who are confronting the immediate implications and consequences of the current outbreak.

In the absence of currently available vaccines, the likely long timeline for vaccine development, and the open questions about the basic pathogenesis of Zika virus infection, parallel development of other prophylactics and therapeutics must be explored. Regarding drugs, the Assistant Director General of the World Health Organization has indicated that preventive therapies, similar to those for malaria, seem like a faster and more workable option than treatments [133]. Currently no small molecule drugs are approved for treatment of Zika infection, although a search of the patent literature reveals many drugs targeting hepatitis C which include claims to Zika virus efficacy. Such antivirals should be evaluated for their efficacy and safety against Zika virus. The anti-malarial hydroxychloroquine is an autophagy inhibitor, and *in vitro* testing has demonstrated inhibition of dengue virus infection *via* induction of reactive oxygen species and mitochondrial antiviral signaling protein [134]. Of interest is that hydroxychloroquine has been safely used during pregnancy [135]. Amodiaquine also acts *via* inhibition of autophagy [136], is safe for use in pregnancy [137], and *in situ* inhibition of Ebola pathogenicity using this compound has been demonstrated at clinically relevant doses [138]. In preliminary cell culture studies, Amodiaquine has also been observed to inhibit the pathogenicity of Zika virus at similar concentrations to those previously reported for Ebola virus (unpublished results by permission, Drs. V Soloveva and S Bavari). Targeted immunotherapeutic strategies may also offer hope for reducing clinical complications from Zika infection including GBS [139, 140], and antibody dependent enhancement (ADE). *In vitro*, ADE has been demonstrated with Zika virus [124]. A US patent issued in 2014, describes a drug useful for treating ADE in dengue that has been verified in *in levitro* and *in vivo* experiments [141].

The potential for monoclonal antibody based therapies for arbovirus infections was recently reviewed [142], concluding that such therapies offer promise as interventions but must be carefully evaluated given the potential challenge of ADE. Engineering to remove Fc binding sequences was shown to mitigate the ADE risk in animal models [143]. Prophylactic and therapeutic use of cross-reactive neutralizing mAbs for flavivirus infections has been shown to be effective in animal models [144]. *De novo* antibodies may be generated which target Zika-specific epitopes. Further study of the role of transplacental immunoglobulin in Zika teratology will be needed.

MAbs which have been appropriately engineered and de-risked have the potential to protect against Zika infection, but a mAb product must have high potency if it is to provide an adequate number of doses at reasonable cost. For example, the adult dose of ZMapp that may reduce the spread of Ebola within the body requires nearly 200 x 10ml vials at 100mg/ml of three antibodies that recognize distinct epitopes of the Ebola Zaire glycoprotein. New tools such as affinity maturation to create a comprehensive map of the paratope sequence space to allow identification of beneficial, neutral, and detrimental amino acid substitutions at each complementarity determining region (CDR) position, as well as use of phage displays, may lead to improved manufacturability (reduced susceptibility to deamidation, oxidation, aggregation) and lead to faster testing of each antibody variant in a cost-effective manner. A similar strategy for Zika, combining two or three mAbs binding non-overlapping specific epitopes, would increase the chances of neutralization by first pass hepatic clearance of the immune complexes. In the absence of dose-response information in humans, a reliable estimate can be obtained from LD50 exposure animal studies where the level of protection may be titrated.

### Outbreak Modeling, Tracking, and Public Health Communications

Zika infection is rapidly spreading throughout the Americas. To keep up with this outbreak, surveillance tracking, outbreak tracking and threat analysis will necessarily involve a combination of methods, both traditional and modern. Traditional methods include case reporting, vector sampling, reservoir animal sampling, and sentinel systems. A number of tools can be added to this list. These include human networking, reporting signature pattern recognition and forecasting, social media tracking and bioinformatics, including geospatial analysis of isolates and immunoinformatics. The West African Ebola virus outbreak of 2014–2015 revealed serious deficiencies in global surveillance, threat identification and management capabilities for infectious disease epidemics. The various lessons-learned exercises which followed may help guide a more effective response to the threats associated with the current outbreak.

Physicians and their patients are asking for practical information to guide routine decisions, and are expressing frustration about public health communication and availability of the clinical tests required to manage important reproductive health decisions. To better understand the questions and issues which medical caregivers and patients need to have addressed, informal on-line surveys were distributed to physicians in the US, Mexico and Argentina. In an initial sample of 56 responses addressing the question “What are the key questions you or your patients might ask about Zika virus?”. The top two responses were “How long does a woman need to wait to get pregnant following potential exposure to Zika virus?” (30%) and “What is the likelihood that a pregnant woman who is exposed to Zika virus will have an infant with a severe defect?” (23%). Many comments focused on frustrations associated with the absence of necessary clinical diagnostic laboratory tests. However, the most telling initial finding involved a question distributed to physicians outside of the United States. In response to “Do you think your health system is prepared for the Zika Virus?”, 79% (343/472) of physicians responded “No”, and 21% (99/472) responded “Yes”.

Prompt and effective public health communications have also been a challenge during both the H1N1 outbreak and the West African Ebola outbreak. In an initiative specifically designed to apply lessons learned from the Ebola experience concerning the importance of rapidly disseminating key information, the International Severe Acute Respiratory and Emerging Infection Consortium (ISARC) in cooperation with Fundação Oswaldo Cruz (Fiocruz), WHO, Institute Pasteur, and the German Centre for Infection Research and others have established an internet-based resource for sharing and developing public health research and response information concerning Zika virus, under the coordination of Fernando Bozza of Fiocruz [145].

PAHO is working to provide timely access to the information which physicians and the public require, and has published a statement on Zika Virus Transmission and Prevention which included the following comments [82]:

- There are two main reasons for the virus's rapid spread: (1) the population of the Americas had not previously been exposed to Zika and therefore lacks immunity, and (2) *Aedes* mosquitoes—the main vector for Zika transmission—are present in all the region's countries except Canada and continental Chile.
- PAHO anticipates that Zika virus will continue to spread and will likely reach all countries and territories of the region where *Aedes* mosquitoes are found.

The National Library of Medicine has established a Disaster Information Research Center website listing resources providing links (https://disaster.nlm.nih.gov/dimrc/zikavirus.html#a6)).

## Conclusions

With the sudden emergence of Zika virus as an evolving epidemic, we are confronted with the need to simultaneously study and understand a new disease, and to develop countermeasures. In many ways Zika presents a much more complex challenge than Ebola, and it may impact more lives. It is vector borne, and therefore its range of transmission will be determined by vector ecosystem. Limiting movement or contact of people cannot significantly contain it. Acute infection may be unapparent, so patients cannot be quarantined. Zika-related disease has its most devastating effects on the unborn fetus with a delay to diagnosis. The transplacental pathology is not understood. The occurrence of GBS suggests that Zika virus associated disease has an autoimmune component. It is epidemic in a region with a high degree of global connectivity; cases will be widely disseminated. The Zika epidemic is moving very rapidly. Research reagents, animal models, and fundamental science knowledge are much less well developed than they were for Ebola. On the other hand, decades of experience with dengue, YFYF, and West Nile have equipped us with familiarity with ADE and flavivirus vaccine development strategies. Zika virus is likely a harbinger of future diseases driven by ecosystem change and global interconnectedness.

Perhaps the biggest challenge with Zika will be to recognize it for what it is: a new disease which does not fit the epidemiology or response paradigm of Ebola or dengue and which will demand effort, resources, unparalleled collaboration, and above all, open mindedness in formulating responses.

**Key Learning Points**

- The pattern of Zika-associated disease observed in Brazil represents a significant public health risk.
- The relationship between infection with Zika virus and primary microcephaly meets most accepted criteria for causality.
- A causal linkage between Zika infection and Guillain–Barré syndrome is plausible, but analysis is complicated by regional co-endemnicity of dengue and chikungunya.
- Possible pathophysiologic interactions between Zika virus infection, microcephaly, other birth defects and GBS are not understood.
- Expedited research will be required to address open questions and to better inform countermeasure development and clinical management.
- Blood banks must promptly implement infection control procedures to secure the supply of critical blood products.
- Methods and policies designed to delay the spread of the virus into uninfected regions will buy critical time to develop medical countermeasures.
- Development of a general use prophylactic vaccine for Zika virus will require considerable time and careful evaluation to mitigate typical vaccine-associated risks in previously healthy unexposed general populations.

**Key Papers in the Field**

Duffy MR, Chen TH, Hancock WT, Powers AM, Kool JL, Lanciotti RS, et al. Zika virus outbreak on Yap Island, Federated States of Micronesia. N Engl J Med. 2009;360(24):2536–43.

Lanciotti RS, Kosoy OL, Laven JJ, Velez JO, Lambert AJ, Johnson AJ, et al. Genetic and serologic properties of Zika virus associated with an epidemic, Yap State, Micronesia, 2007. Emerg Infect Dis. 2008;14(8):1232–9.

Musso D, Nhan T, Robin E, Roche C, Bierlaire D, Zisou K, et al. Potential for Zika virus transmission through blood transfusion demonstrated during an outbreak in French Polynesia, November 2013 to February 2014. Euro Surveill. 2014;19(14).

Faye O, Freire CC, Iamarino A, Faye O, de Oliveira JV, Diallo M, et al. Molecular evolution of Zika virus during its emergence in the 20(th) century. PLoS Neglected Tropical Diseases. 2014;8(1):e2636.

Musso D, Roche C, Robin E, Nhan T, Teissier A, Cao-Lormeau VM. Potential sexual transmission of Zika virus. Emerg Infect Dis. 2015;21(2):359–61.

Hamel R, Dejarnac O, Wichit S, Ekchariyawat P, Neyret A, Luplertlop N, et al. Biology of Zika Virus Infection in Human Skin Cells. J Virol. 2015;89(17):8880–96.

Oliveira Melo AS, Malinger G, Ximenes R, Szejnfeld PO, Alves Sampaio S, Bispo de Filippis AM. Zika virus intrauterine infection causes fetal brain abnormality and microcephaly: tip of the iceberg? Ultrasound Obstet Gynecol. 2016;47(1):6–7.

Mlakar J, Korva M, Tul N, Popović M, Poljšak-Prijatelj M, Mraz J, et al. Zika Virus Associated with Microcephaly. N Engl J Med. 2016 Feb 10.
